# Pattern of expression of vascular endothelial growth factor and its receptors in the ovine choroid plexus during long and short photoperiods

**DOI:** 10.1007/s00441-012-1431-7

**Published:** 2012-05-24

**Authors:** Aleksandra Szczepkowska, Barbara Wąsowska, Przemysław D. Gilun, Christine Lagaraine, Vincent Robert, Laurence Dufourny, Jean-Claude Thiéry, Janina Skipor

**Affiliations:** 1 Institute of Animal Reproduction and Food Research, Polish Academy of Sciences, Olsztyn, Poland; 2 INRA, UMR85 Physiologie de la Reproduction et des Comportements, F-37380 Nouzilly, France; 3 CNRS, UMR 6175, F-37380 Nouzilly, France; 4 Université de Tours, F-37041 Tours, France; 5 Haras Nationaux, F-37380 Nouzilly, France

**Keywords:** VEGF, VEGF receptors, Choroid plexus, Photoperiod, Sheep

## Abstract

Vascular endothelial growth factor (VEGF-A) plays an important role in maintaining cerebrospinal fluid (CSF) homeostasis and the function of the choroid plexuses (CPs). The objective of the study was to determine the expression of vascular endothelial growth factor (VEGF-A), tyrosine kinase receptors Flt-1 and KDR and KDR co-receptor neuropilin 1 (NRP-1) in ovine CPs during different photoperiods. CPs were collected from the lateral brain ventricles from ovariectomized, estradiol-treated ewes during long day (LD; 16L:8D, *n* = 5) and short day (SD; 8L:16D, *n* = 5) photoperiods. We analyzed mRNA expression levels of two VEGF-A isoforms, *VEGF-A*_*120*_ and *VEGF-A*_*164*_ and our results indicate that *VEGF-A*_*164*_ was the predominant isoform. Expression levels of *VEGF-A* and *Flt-1* were similar during the SD and LD photoperiods. There were significant increases in *KDR* mRNA and protein expression (*p* < 0.05) and *NRP-1* mRNA expression (*p* < 0.05) during SD. These data show that expression of KDR and its co-receptor NRP-1 are up-regulated by short photoperiod and that this effect is not dependent on ovarian steroids. Our results suggest that the VEGF-A-system may be involved in photoperiodic plasticity of CP capillaries and may therefore be responsible for photoperiodic changes in the CSF turnover rate in ewes.

## Introduction

The choroid plexuses (CPs) are located in the ventricles of the vertebrate brain. They are formed by a monolayer of epithelial cells surrounding a central stroma in which blood vessels with fenestrated endothelium are embedded in an extracellular matrix (Redzic and Segal 2004). CPs are involved in the basic aspects of neural function, including maintenance of the extracellular milieu of the brain by secreting cerebrospinal fluid (CSF) and active modulation of substance exchange between the CSF and blood plasma, as well as removal of metabolic products from the brain (Cserr 1971; Chodobski et al. 1998; Skipor and Thiery 2008). Therefore, even modest changes in the organization of CPs may cause variability in the composition of CSF. Variability in the CSF makeup seems to influence changes in brain activity, which may affect behavioral states and neuroendocrine events (Veening and Barendregt 2010). In a previous study, we demonstrated that different photoperiodic statuses were associated with severe modulations of progesterone and estradiol concentrations in the CSF of ewes (Thiéry et al. 2003, 2006). These photoperiod-driven changes in the CSF steroid content require the presence of the pineal gland (Thiéry et al. 2006) and illustrate the probable involvement of CSF and CPs in the neuroendocrine regulation of seasonal reproduction (Thiéry and Malpaux 2003). However, the cellular mechanisms underlying these changes in CSF hormonal concentrations remain unknown. On the one hand, recent work has demonstrated photoperiodic changes in tight junction protein expression in CPs (Lagaraine et al. 2011). On the other hand, these differences in hormonal content may be explained by variations in the CSF secretion rate with photoperiod, which would result in concentration or dilution of hormone molecules. We demonstrated that the turnover rate (TOR) of CSF in ewes is higher on short days (SD) than on long days (LD) (Thiéry et al. 2009).

CSF homeostasis and CP function are maintained by numerous factors, including vascular endothelial growth factor (VEGF-A), which is continuously and highly expressed in the CPs and plays an important role in regulating the stability of the endothelial cells in the CPs (Maharaj et al. 2008). VEGF-A is involved in maintaining endothelial cells’ fenestrated phenotype in the CP capillaries (Esser et al. 1998; Roberts and Palade 1995). VEGF-A belongs to the VEGF family of proteins, which, in humans, also contains VEGF-B, VEGF-C, VEGF-D and placental growth factor (Bates 2010). VEGF-A is a homodimeric glycoprotein of 40–45 kDa and several isoforms exist as a result of mRNA alternative splicing. In humans, there are at least six splice variants of *VEGF-A*, encoding 121, 145, 165, 183, 189 and 206 amino acid proteins (Robinson and Stringer 2001). In sheep, these isoforms are one amino acid shorter due to a deletion in the N-terminal region (Tischer et al. 1991). The isoforms share the same function and the main difference between them lies in their ability to bind heparin (Gitay-Goren et al. 1992). The differential heparin-binding properties are related to the bioavailability of the isoforms (Houck et al. 1992). The smaller isomers (121, 145, or 165 amino acids) are secreted from cells, whereas VEGF-A_189_ and VEGF-A_206_ are almost completely sequestered in the extracellular matrix (Houck et al. 1991, 1992; Ashikari-Hada et al. 2005; Ruhrberg et al. 2002). The effects of VEGF-A are transduced mainly by two high-affinity receptors belonging to the tyrosine kinase family: the fms-like tyrosine kinase (Flt-1) and the fetal liver kinase-1/kinase insert domain-containing receptor (Flk-1/KDR). VEGF-A_165_ but not VEGF-A_121_, also binds with neuropilin-1 and -2 (NRP-1 and NRP-2), which have been identified in endothelial cells (Gluzman-Poltorak et al. 2000, 2001; Soker et al. 1998). Unlike Flt-1 and Flk-1/KDR, NRP-1 and -2 do not have a tyrosine kinase domain and, therefore, cannot induce cellular responses alone.

CPs have been shown to contain mRNA (Nico et al. 2004) and protein (Maharaj et al. 2008; Witmer et al. 2002; Yang et al. 2010) encoding the VEGF-A receptors. Most of the information on the localization and expression of VEGF-A has been derived from studies on rodents. No data are available concerning expression of components of the VEGF-A system in ovine CPs. Therefore, to gain insight into the possible involvement of VEGF-A in regulating differential hormonal concentrations in CSF in accordance with photoperiod, we studied the expression of VEGF-A system components during artificial SD and LD.

## Materials and methods

### Animals and treatment

The studies were performed on four ewes of the Polish Lowland breed (3–4 years old, 50–60 kg weight) housed in a natural SD photoperiod (September, at 52°N, 21°E) and ten adult (3 years old, 50–60 kg weight), ovariectomized Ile-de-France ewes (the same as those used in the study by Lagaraine et al. 2011) implanted with estradiol (E_2_) and kept in artificial lighting conditions including LD (16L:8D, *n* = 5) and SD (8L:16D). The LD group was transferred from natural light at the end of August to SD for 4 months and then to LD for 90 days. The SD group was maintained under LD for 4 months from the end of August and then switched to SD for 90 days. The E_2_ implants, inserted at the same time as the ovariectomy (directly before transfer to artificial light conditions), were made from Silastic tubing; they maintained plasma E_2_ concentrations of 2–4 pg/ml (Thiéry et al. 2006). The schedule of light stimulation was described in detail by Lagaraine et al. (2011). To assess responsiveness to the photoperiodic treatments, blood samples were collected from the jugular vein twice weekly for 2 weeks before animal slaughter and plasma luteinizing hormone (LH) concentrations were subsequently assayed. Animals were killed by a licensed butcher in a certified slaughterhouse. All Polish Lowland ewes were in follicular phase of the estrous cycle, determined by examination of the morphology of the ovaries. After decapitation, the brains of 10 sheep housed in artificial lighting conditions were dissected. CPs were removed from their anchoring to the Galien’s vein and the split was made along the mid-line, separating the CP from each lateral ventricle (one part for mRNA and one part for protein assay). CPs were then immediately frozen in liquid nitrogen and stored at −80 °C until use. Studies were conducted in accordance with the Polish Guide for the Care and Use of Animals (1997) and approved by the Local Ethics Committee (agreement no. 4/2008) as well as with French Authorization No.37801 for Animal Experimentation and Surgery from the French Ministry of Agriculture, following the European Community Council Directive 86/609/EEC. Indoor light treatments, surgery and postoperative care were conducted in certified facilities (ISO9001/2000 version, July 2006, No. B37-175-2).

### LH measurements

Plasma LH concentrations were determined using the double antibody ELISA immunoassay technique, as previously described (Faure et al. 2005). The intra- and inter-assay coefficients of variation of the control averaged 12 and 8 %, respectively. The minimum detectable concentration for LH was 0.1 ng/ml.

### Tissue collection and preparation for immunohistochemistry

Immediately after decapitation, brains from four sheep (housed in conditions with natural photoperiods) were perfused via both carotids with 1,500 ml of 0.1 M phosphate buffer (PB, pH 7.4) and subsequently with 1,500 ml 0.1 M PB containing 4 % (w/v) paraformaldehyde, pH 7.4. The brain tissue surrounding the ventricles with CPs was removed 20 min after perfusion began, post-fixed for 24 h in the same fixative and washed with 0.01 M PB. Brain fragments joined with the CPs were cryoprotected in a 20 % sucrose solution in 0.1 M PB with 0.1% sodium azide at 4 °C for at least 7 days and then kept at −40 °C until further processing. Frozen tissues were cut using a cryostat (Leica CM 3000, Germany) into 10-μm-thick sections and placed onto silane-coated slides (3-aminopropyltriethoxysilane; Sigma). Sections were briefly air-dried and washed 3 times with 0.1 M phosphate-buffered saline (PBS; pH 7.4). The remaining steps were carried out at room temperature (RT).

### Immunohistochemistry

Sections were incubated for 30 min with 3 % hydrogen peroxide in PBS to quench endogenous peroxidase activity. After 3 washes (10 min each) in PBS, they were incubated for 60 min in a blocking solution (BS) containing the following: PBS with 10 % normal goat serum (NGS; Sigma), 0.1 % Triton X-100 (ICN Biomedicals, USA), 0.2 % bovine serum albumin (BSA; ICN Biomedicals) and 0.05 % Thimerosal (Sigma). A primary antibody raised against VEGF (A-20, sc-152, rabbit polyclonal; working dilution 1:30–1:50; Santa Cruz Biotechnology, USA) was diluted in BS and applied to sections overnight. Following subsequent rinsing with PBS (3 × 10 min), the sections were incubated for 60 min with Alexa Fluor 594 goat anti-rabbit secondary antibody (working dilution 1:100; Invitrogen, Molecular Probes, USA) in BS to visualize the anti-VEGF antibody. Sections were then washed in PBS and coverslipped with a mounting medium for fluorescence containing 4’,6-diamidino-2-phenylindole dihydrochloride (DAPI; Vector Laboratories, CA, USA) to visualize cellular nuclei. Control sections omitting the primary antibody and both primary and secondary antibodies were processed for each labeling reaction to detect non-specific binding of the antibody and autofluorescence, respectively (data not shown). Positive controls were prepared by treating sections of the VEGF-immunoreactive porcine and ovine endometrium and porcine umbilical cord with the primary and secondary antibodies at the same dilutions used for the experimental tissues (data not shown). The sections were viewed using an automated Zeiss Axio Imager.Z1 upright microscope fitted with a Zeiss Axiocam MRm digital monochrome CCD camera (Carl Zeiss Vision) with Apotome AxioVision Rel. 4.8 program (Zeiss) (emission filter: DAPI – 461 nm, Alexa Fluor 594 – 617 nm; excitation filter: DAPI – 358 nm, Alexa Fluor 594 – 590 nm).

### Gene expression assays

One part of CP from each animal was cut into small pieces and 20 mg of frozen tissue was homogenized in Qiazol lysis reagents (Qiagen) in Lysing Matrix D (MP Biomedicals, Illkrich, France) with a FastPrep-24 instrument (MP Biomedicals). Total RNA was extracted using the RNeasy lipid tissue mini kit (Qiagen) and DNase 1 (Qiagen) to eliminate possible genomic DNA contamination according to the manufacturer’s instructions. The concentration and quality of RNA isolated from the CP tissue were determined using a NanoDrop (Thermo Scientific) and 2 % agarose gel electrophoresis. Two micrograms of total RNA was saved for further use in RT (reverse transcription) reactions. RT reactions were performed with a total reaction volume of 40 μl containing AMV Reverse Transcriptase, RNase Inhibitor, Oligo (dT) primers and dNTPs mixed at the concentrations suggested in the protocol supplied by the manufacturer (Promega). The resulting cDNA was diluted in nuclease-free water (Promega) and stored at −20 °C until further analysis.

The VEGF isoform content in ovine CPs was determined using RT-PCR with primers designed to amplify all isoforms of *VEGF-A* (Table 1) using REDTaq Ready Mix (Sigma). The following protocol was used: 95 °C for 7 min for hot start REDTaq DNA polymerase; followed by 35 cycles of 95 °C for 20 s (denaturation), 55 °C for 20 s (annealing), 72 °C for 20 s for extension and, finally, 72 °C for 7 min (last chain elongation). After PCR was performed, the products were separated on 4 % agarose gels, treated with 0.01 % ethidium bromide and examined under UV light (Gel Logic100; KODAK).

**Table 1:** Sequences of oligonucleotide primers used for RT-PCR and qRT-PCR analyses

Gene	Primers (5' → 3')	Product size	Reference
*VEGF-A* all isoforms^a^	Forward: TGCGGATCAAACCTCACCAAA	125–380 bp	Tsoi et al. (2002)
	Reverse: TCACCGCCTCGGCTTGTCACA		
*VEGF-A*_*120*_	Forward: AAGGCCAGCACATAGGAGAG	101 bp	Kaczmarek et al. (2008)
	Reverse: CCTCGGCTTGTCACATTTTT		
*VEGF-A*_*164*_	Forward: GAGGCAAGAAAATCCCTGTG	150 bp	Kaczmarek et al. (2008)
	Reverse: TCACATCTGCAAGTACGTTCG		
*Flt-1* VEGF receptor 1	Forward: TGGATTTCAGGTGAGCTTGGA	68 bp	Redmer et al. (2005)
	Reverse: TCACCGTGCAAGACAGCTTC		
*KDR* VEGF receptor 2	Forward: CTTCCAGTGGGCTGATGACC	67 bp	Redmer et al. (2005)
	Reverse: GCAACAAACGGCTTTTCATGT		
*NRP-1* neuropilin	Forward: GATTGCGGTGGACGATATTAGC	60 bp	Vonnahme et al. (2006)
	Reverse: GGTTTTGCGCAGTCCTCTTG		
*PPIC* peptidyl-prolyl cis-trans isomerase C	Forward: TGGCACTGGTGGTATAAGCA	145 bp	Herman et al. (2010)
	Reverse: GGGCTTGGTCAAGGTGATAA		

^a^Primers used in RT-PCR analysis, the expected sizes for ovine VEGF205, 188, 164, 145 and 120 are 380, 329, 257, 198 and 125 bp, respectively.

To further evaluate the effects of photoperiod on mRNA expression of components of the VEGF-A-receptor system, real-time PCR was performed on cDNA prepared from ovine mRNA isolated from CPs collected from animals subjected to SD and LD photoperiods. Analyses were performed with an ABI Prism 7900 sequence detection system using Power SYBR green PCR master mix (Applied Biosystems by Life Technologies, Carlsbad, CA, USA). Specific primer pairs for the different genes were used according to literature (Table 1). All primers were synthesized by IBB PAN (Poland). PCR-derived DNA fragments (*VEGF-A*_*120*_ *, VEGF-A*_*164*_ *, Flt-1, KDR, NRP-1*) were separated by electrophoresis on 2 % agarose gels supplemented with 0.01 % ethidium bromide and examined under UV light (Gel Logic100; KODAK). *VEGF-A*_*120*_ and *VEGF-A*_*164*_ PCR products were sequenced (Oligo IBB PAN; Poland) to confirm their specificity for sheep.

Each real-time PCR reaction well (20 μl) contained 2 μl of diluted RT product, 0.2 μM forward and reverse primers each and 10 μl of Power SYBR green PCR master mix. The following protocol was used: 95 °C for 15 min for Hot Start AmpliTaq Gold DNA polymerase and 38 cycles of 95 °C for 10 s (denaturation), 55 °C for 20 s (annealing) and 72 °C for 20 s (extension). After the cycles, a final melting curve analysis under continuous fluorescence measurement was performed to evaluate the specific amplification. The results were analyzed using Real-time PCR Miner (on-line available: http://www.miner.ewindup.info/version2), based on the algorithm developed by Zhao and Fernald (2005).

### SDS-PAGE and immunoblotting

The second part of CP was cut into small pieces, placed frozen into lysing Matrix D tubes (MP Biomedicals, Solon, OH, USA) with 500 μl of ice-cold lysis buffer consisting in 100 mM NaCl, 1 % Triton X-100, 2 mM EDTA, 0.2 % SDS, 0.5 % sodium deoxycholate and 1 % protease inhibitor cocktail and homogenized in the FastPrep instrument (MP Biomedicals) at an oscillation speed of 6.5 for 30 s. Disruption was repeated 3 times and between the cycles, samples were placed on ice. After the last cycle, tubes were briefly centrifuged at 5,000*g* to remove any undisrupted tissues. Homogenates were then transferred into new tubes and centrifuged at 13,000*g* for 30 min at 4 °C. The obtained supernatants were used for protein quantification using a Bradford kit (Uptima kit; Interchim, Montluçon, France). Aliquots of 100 μg of protein were stored at −20 °C until being loaded on SDS-polyacrylamide gradient gels (6–15 % for Flt-1 and KDR and 6–12 % for VEGF-A) and then transferred to a 0.45-μm PVDF (Millipore, Billerica, MA, USA) membrane using wet (VEGF-A) or semi-dry (Flt-1, KDR) techniques. Molecular weight standards were included for each immunoblot. The membranes were then blocked with 5 % non-fat milk in TBST (Tris-buffered saline with 0.5 % Tween-20) buffer for 1.5 h at room temperature, extensively washed in TBST and incubated overnight at 4 °C with the appropriate primary antibody solution. The following primary antibodies were used: rabbit polyclonal anti Flt-1 (C-17; 1:40), rabbit polyclonal anti-Flk-1 (C-20; 1:40), rabbit polyclonal anti-VEGF-A (A-20; 1:200) (all from Santa Cruz Biotechnology). VEGF-A immunoblots were washed in TBST 3 times and then incubated for 1.5 h at room temperature with goat anti-rabbit alkaline phosphatase-conjugated polyclonal antibodies (Sigma-Aldrich, St. Louis, MO, USA) at a dilution of 1: 20,000. Binding of the secondary antibody was visualized with NBT/BCIP solution (Sigma). Next, the blots were examined under white light (Gel Logic100; KODAK). The Flt-1 and KDR immunoblots were incubated for 1.5 h at room temperature with goat anti-rabbit biotin-conjugated antibodies included in the WesternDot™ kit (Invitrogen by Life Technologies, Carlsbad, CA, USA) and visualized with Qdot 625 streptavidin conjugate (Invitrogen by Life Technologies) according to the manufacturer’s instructions. Then, the blots were examined under UV light (Gel Logic100; KODAK). The blots were stripped and re-probed with rabbit polyclonal anti-GAPDH antibody conjugated to horseradish peroxidase (Santa Cruz Biotechnology) as the protein loading control, which was then detected with the enhanced chemiluminescence SuperSignal^®^ West Dura Kit (Thermo Scientific, West Palm Beach, FL, USA) and imaged with G BOX iChemi XT (SYNGENE, Cambridge, UK). Additionally, some blots were incubated with antibodies pre-adsorbed with excess amounts of their respective peptides (Flt-1 (C-17)P, Flk-1 (C-20)P, VEGF-A (A-20)P; Santa Cruz Biotechnology). To identify VEGF-A isoforms, western blot analyses were performed using recombinant human (rh) VEGF-A_121_ and rhVEGF-A_165_ isoforms (PeproTech, UK).

### Data analysis

The real-time PCR results are presented as the relative gene expression of the target gene vs. the housekeeping gene (*PPIC*). The western blot results are presented as arbitrary units of optical density of the target proteins normalized to GAPDH protein as a loading control. Values represent the mean ± SEM for each group (short and long photoperiods). The significance of differences between the SD and LD groups was assessed by the Mann–Whitney *U* test (PRISM 4, Graph Pad, USA).

## Results

### Responsiveness to the photoperiodic treatments

In the LD group, LH was not detected in all five animals (<0.1 ng/ml), whereas in the SD group, the LH levels ranged from 2.7 ± 0.4 to 3.7 ± 0.7 ng/ml (mean ± SEM, data not shown), which is in accordance with expectations for this experimental model (Goodman et al. 1982). These data have been reported in our previous article (Lagaraine et al. 2011).

### Immunohistochemistry

**Fig. 1:**
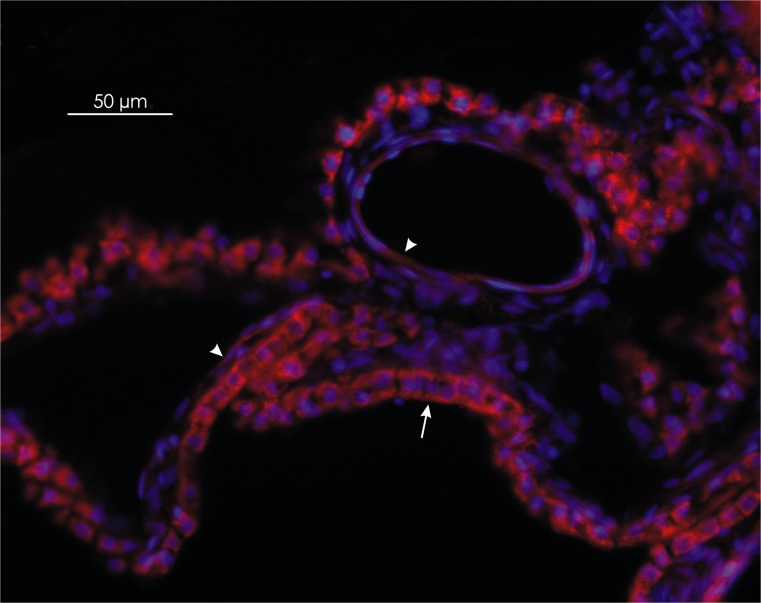
Distribution of vascular endothelial growth factor (VEGF-A) in the ovine choroid plexus. The red immunoreactivity of VEGF-A is strongly expressed in cobblestone-shaped epithelial cells (*arrow*), in comparison with the weaker immunoreactive signal observed in the spindle-shaped endothelial cells (*arrowheads*). Magnification ×200

Immunohistochemical staining with a polyclonal antibody against VEGF-A that detects the three isoforms (VEGF-A_121_, VEGF-A_165_ and VEGF-A_189_) showed labeling for VEGF-A in epithelial and endothelial cells of CPs (Fig. 1). However, the immunoreactivity in epithelial cells was stronger than in endothelial cells.

### Effect of photoperiod on mRNA expression for VEGF-A and its receptors

**Fig. 2:**
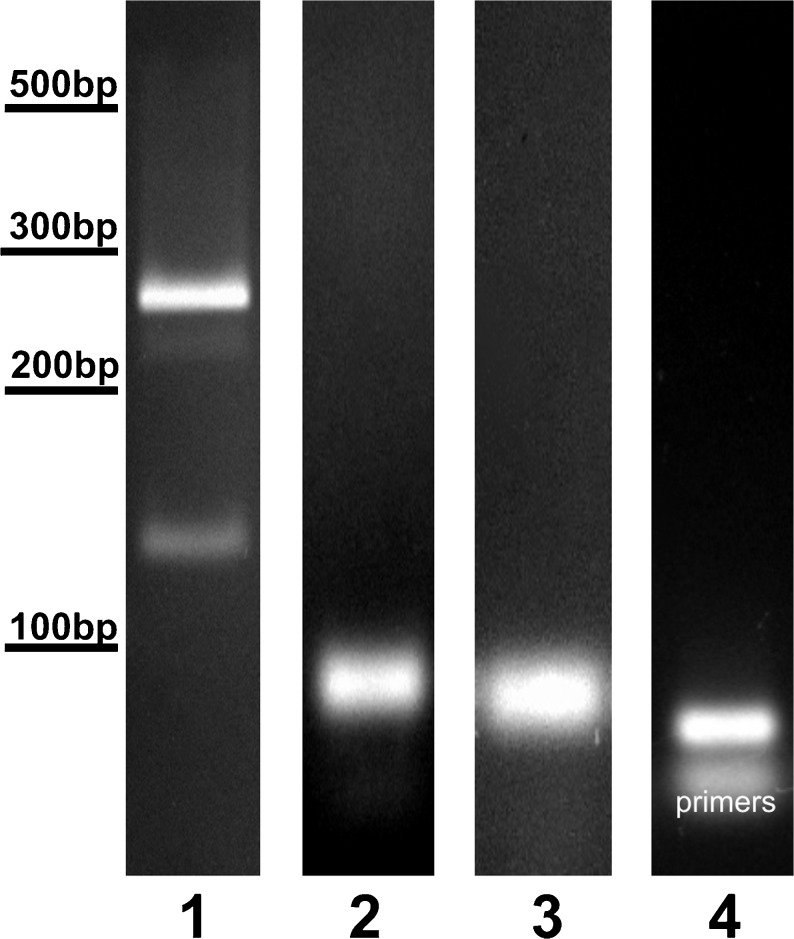
Representative photographs of RT-PCR products. Two isoforms of VEGF-A (*line 1*) were found in choroid plexus samples, *VEGF-A*_*164*_ (275 bp) and *VEGF-A*_*120*_ (125 bp). Single transcripts for *Flt-1* (68 bp) (*line 2*), *KDR* (145 bp) (*line 3*) and *NRP-1* (60 bp) (*line 4*) were observed

**Fig. 3:**
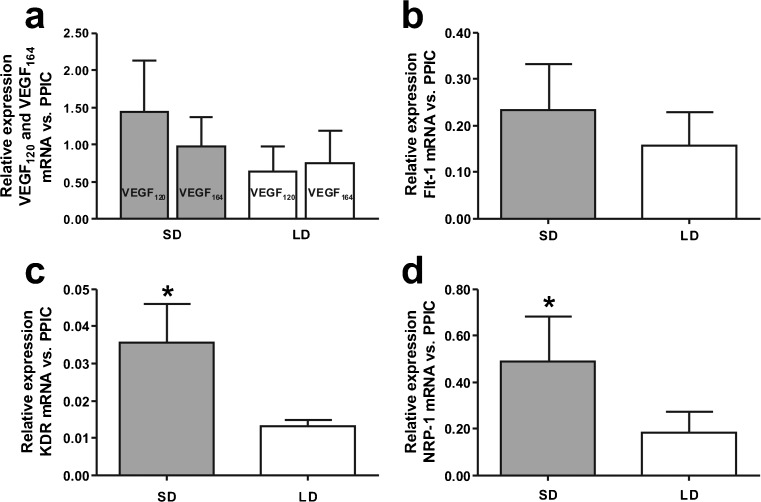
Expression of *VEGF-A*_*120*_ and *VEGF-A*_*164*_ isoforms (**a**), *Flt-1* (**b**), *KDR* (**c**) and *NRP-1* (**d**) mRNA levels in the ovine choroid plexuses during short days (SD; 8L:16D) and long days (LD; 16L:8D). All values are presented as the mean ± SEM of ratios relative to *PPIC* determined by real-time PCR (*n* = 5 /SD or LD photoperiod). *p < 0.05

RT-PCR experiments performed on mRNA isolated from ovine CPs showed two *VEGF-A* products corresponding to the *VEGF-A*_*120*_ and *VEGF-A*_*164*_ isoforms (Fig. 2, line 1). The relative abundance of these isoforms was as follows: *VEGF-A*_*164*_ > *VEGF-A*_*120*_. Real-time PCR analysis demonstrated similar levels of mRNA expression between the SD and LD photoperiods for *VEGF-A*_*120,*_ *VEGF-A*_*164*_ (Fig. 3a) and *Flt-1* (Fig. 3b). However, the expression of *KDR* (Fig. 3c) *and NRP-1* (Fig. 3d) was significantly higher (*p* < 0.05) during SD than LD.

### Effect of photoperiod on protein expression of VEGF-A and its receptors

**Fig. 4:**
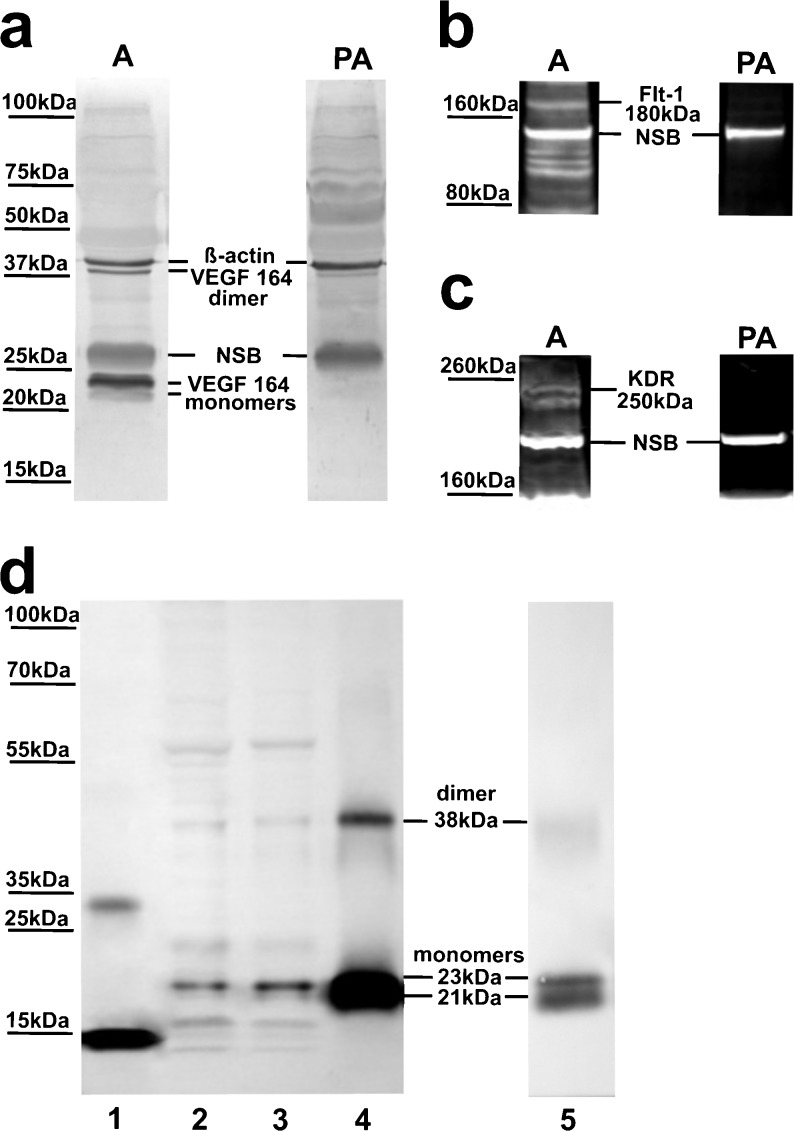
Specificity of the VEGF-A (**a**), Flt-1 (**b**) and KDR (**c**) antibodies in western blot analyses of ovine choroid plexus (CP) homogenates. Samples were probed with either free antibody or antibody pre-adsorbed with specific control peptides. **d** Representative blots of recombinant human (rh)VEGF-A_121_ (*line 1*), ovine CPs (*lines 2 and 3*) and rhVEGF-A_165_ (*lines 4 and 5*) resolved by SDS-PAGE and immunoblotted with VEGF-A antibodies used in (**a**). Blots in (**a**) were re-probed with β-actin antibodies.* A* antibodies,* PA* pre-adsorbed antibodies, *NSB* non-specific binding

**Fig. 5:**
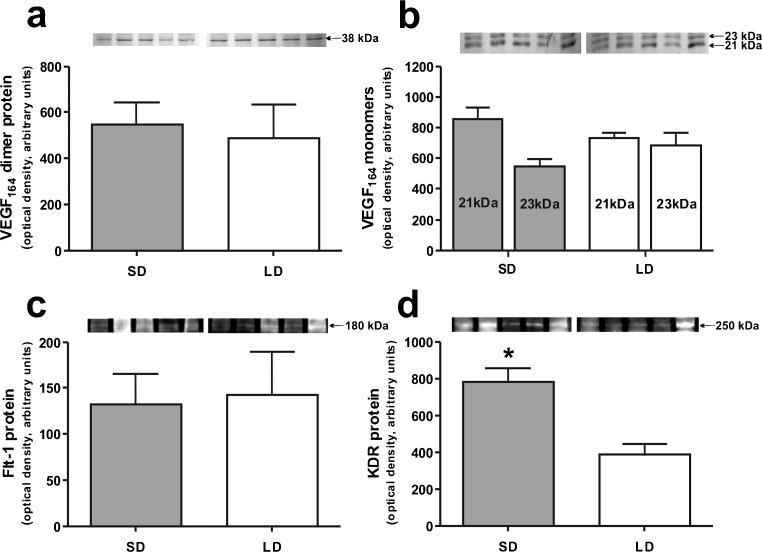
Western blot analyses of VEGF-A: VEGF-A_164_ dimer (**a**), VEGF-A_164_ monomers 21 and 23 kDa (**b**), Flt-1 (**c**) and KDR (**d**) in ovine choroid plexuses (CPs) during short days (SD; 8L:16D) and long days (LD; 16L:8D). *Upper panels* representative blots of CPs resolved by SDS-PAGE and immunoblotted with VEGF-A, Flt-1 and KDR antibodies. VEGF-A was visualized with alkaline phosphatase, whereas Flt-1 and KDR were visualized using the WesternDot™ 625 Western Blot Kit, which requires UV light for visualization.* Lower panels* mean ± SEM of the densitometric analysis of relative protein levels. *p < 0.05

Under our experimental conditions, VEGF-A migrated on SDS-PAGE gels both as a dimer of approximately 38 kDa and monomers of ~21 and 23 kDa (Fig. 4a), which corresponded to rhVEGF-A_165_ dimer and monomers (Fig. 4d). The protein levels of VEGF-A_164_ (dimer and monomers) were similar in the SD and LD photoperiods (Fig. 5a, b).

Specific bands at approximately 180 kDa and 250 kDa in CP samples were observed when antibodies for Flt-1 and KDR were used, respectively (Fig. 4b, c). Flt-1 protein levels were similar in both photoperiods (Fig. 4c), whereas the expression of KDR protein was significantly higher (*p* < 0.05) during SD than LD (Fig. 4d).

## Discussion

For the first time, we demonstrated that photoperiod affects the expression of the VEGF-A-receptor system in ovine CPs. Using RT-PCR, we confirmed that two *VEGF-A* isoforms, *VEGF-A*_*120*_ and *VEFG-A*_*164*_, are expressed in ovine CPs and that *VEGF-A*_*164*_ is the predominant isoform in this structure. This is in agreement with a study by Esser et al. (1998) that demonstrated that mRNA of *VEGF-A*_*164*_ and *VEGF-A*_*120*_ isoforms are expressed in bovine CPs. Of note, in mouse CPs, an additional isoform, *VEGF-A*_*188,*_ is also present in very limited amounts (1.7 %) compared with *VEGF-A*_*120*_ (52.6 %) and *VEGF-A*_*164*_ (45.6 %) forms (Maharaj et al. 2008). This suggests that the mechanisms of alternative splicing are different between sheep and mice in the CPs. Our RT-PCR results were confirmed by protein content analyses. Immunoblots demonstrated the presence of VEGF-A_164_ dimer (38 kDa) and monomer (~21 and 23 kDa) forms, which were similar to the rhVEGF-A_165_ we used. In reducing conditions, VEGF-A_165_ migrated as a doublet of 21 and 23 kDa, probably as unglycosylated and glycosylated forms (Houck et al. 1991). We did not find VEGF-A_120_ proteins in CP homogenates, which may stem from the biochemical nature of this isoform. VEGF-A_121_ is a non-heparin-binding acidic protein, which is freely released from producing cells, whereas 50–70 % of VEGF-A_165_ remains in cells and the associated extracellular matrix (Houck et al. 1992). The differential affinity for heparan sulfate is important for binding of VEGF-A isoforms to VEGF-A receptors because heparan sulfate can mediate in the binding and transactivation of these receptors (Selleck 2006). Furthermore, differential binding to heparan sulfate is reported to lead to different VEGF-A actions, including endothelial cell survival, adhesion and vascular branch formation (Ashikari-Hada et al. 2005; Ruhrberg et al. 2002).

Both VEGF-A isoforms are presumably secreted in both directions, into the CSF, as VEGF-A is detectable in normal CSF (Schänzer et al. 2004) and toward endothelial cells in CPs. Immunohistochemical staining was performed with an antibody against VEGF-A that detects three different isoforms of 121, 165 and 189 amino acids. This analysis demonstrated that VEGF-A is expressed in both the epithelial and endothelial cells of CPs. VEGF-A is synthesized in epithelial cells of the CPs (Maharaj et al. 2006), so staining of endothelial cells may therefore represent secreted VEGF-A isoforms. Numerous studies have demonstrated that VEGF-A secreted from epithelial cells of CPs exerts a paracrine action on Flt-1 and KDR receptors found on endothelial cells (Nico et al. 2004; Maharaj et al. 2008). In addition to *Flt-1* and *KDR* mRNA, we demonstrated in ovine CPs expression of mRNA for *NRP-1,* which may indirectly confirm VEGF-A_164_ action on CP endothelial cells.

The capillaries of CPs are VEGF-dependent. Endothelial fenestrations and high expression of KDR and Flt-4 (VEGFR3), are markers of this feature (Partanen et al. 2000; Kamba et al. 2006). VEGF-dependent capillaries have phenotypic plasticity whereby some may undergo regression and others lose fenestrations, down-regulate KDR and Flt-4 and survive by becoming insensitive to VEGF inhibition (Kamba et al. 2006). For example, treatment of adult mice with a VEGFR tyrosine kinase inhibitor (AG-013763) for 3 weeks resulted in CP capillary regression by 45 % (Kamba et al. 2006). Moreover, the organ-specific differences in the sensitivity of fenestrated capillaries to VEGF inhibition and rapid re-growth of capillaries after cessation of inhibition found in that study suggest multiple levels of vascular plasticity in response to changes in local concentrations of VEGF. In the current study, we demonstrated that in ovine CPs, expression of KDR and NRP-1 but not VEGF-A and Flt-1, is regulated by the photoperiod. Exposure of ewes to SD conditions significantly increased the mRNA and protein levels of KDR and mRNA levels of *NRP-1*. Taken together, these results suggest that the VEGF-A-system is involved in photoperiodic plasticity of CP capillaries and may be responsible for photoperiodic changes in TOR of CSF in sheep (Thiéry et al. 2009). To date, descriptions of seasonal alterations in brain vasculature have been limited to birds. In adult male canaries, increased circulating testosterone concentrations that are characteristic of the breeding season have been described to induce VEGF production and microvascular expansion in the higher vocal center of the brain (Louissaint et al. 2002). Interestingly, the first photoperiod-evoked changes in adult brain angiogenesis in mammals were described by Pyter (2006) in her doctorate thesis. She reported that *VEGF* expression in some regions of the brain in male white-footed mice was photoperiodically regulated. She showed that acute transfer of mice from SD to LD decreased hippocampal and olfactory bulb *VEGF* expression.

At present, the mechanisms responsible for photoperiodic modulation of KDR and NRP-1 expression in ovine CPs are not known. A study by Kremer et al. (1997) demonstrated that VEGF-A_165_ can up-regulate KDR expression in the endothelial cells of cultured brain slices. In our studies, we did not observe increased amounts of VEGF-A_164_ protein in the CPs during SD. However, this may be due to the secretory nature of this isoform. The expression of mRNA for both *VEGF-A* isoforms was slightly higher in SD than LD but this difference was not statistically significant due to the high variability in the SD group. Taking into account the hormonal status of ewes in both photoperiods, we can exclude the effect of altered concentraction of ovarian steroids on KDR and NRP-1 expression in the CPs because all ewes were ovariectomized and E2 treated, allowing the maintenance of constant E2 levels in the SD and LD groups (Thiéry et al. 2006). The action of higher concentration of E2 found by Thiéry et al (2006) in the CSF during LD (14.9 ± 2.8 pg/ml) than SD (9.4 ± 1.7 pg/ml) may also be excluded as a cause of the differences because CP cells were under the influence of a constant value of E2 in general circulation. In mammals, photoperiod is considered to be the most important factor entraining the circannual physiological rhythms through changing circadian patterns of melatonin secretion from the pineal gland (Reiter 1991). A short day length results in an extended duration of nocturnal melatonin secretion. High concentrations of melatonin in the CSF (Skinner and Malpaux 1999) may act on CPs because mRNA expression of the melatonin receptors *MT1* and *MT2* has been demonstrated in ovine CPs (Cogé et al. 2009). In sheep, photoperiod or melatonin has been described to modulate the concentration of steroids (Thiéry et al. 2003, 2006), leptin (Adam et al. 2006) and triiodothyronine (Skipor et al. 2010) in the CSF. Melatonin also stimulates the secretory activity of CPs in hamsters and rats (Decker and Quay 1982; Vitte et al. 1989). Taken together, these data suggest that melatonin may be responsible for the up-regulation of KDR and NRP-1 expression in ovine CPs under SD. However, the possible involvement of melatonin in the regulation of the VEGF-A system expression and, consequently, in the plasticity of CP capillaries remains to be ascertained.

In conclusion, we demonstrated that VEGF-A has two splice variants encoding isoforms of 120 and 164 amino acids and VEGF-A_164_ is the predominant isoform in ovine CPs. Expression of both KDR and NRP-1 was higher during SD than LD, whereas expression of VEGF-A and Flk-1 was not affected. Therefore, this is the first study demonstrating photoperiodic changes in the VEGF-A system in the normal adult brain vasculature in domestic animals. Future studies are needed to better understand the functional and mechanistic aspects of photoperiod modulation of the CP structure.
